# Uncommon presentations of type A quadricuspid aortic valve in the Septuagenarian

**DOI:** 10.1186/s13019-024-02696-w

**Published:** 2024-05-29

**Authors:** Perry Choi, Michael Paulsen, Yihan Lin, William Moskalik, Angela Ji, Ethan Jackson, Sachin Malik, Elan Burton, Y Joseph Woo, Thomas Burdon

**Affiliations:** 1 Department of Cardiothoracic Surgery, Stanford University Medical Center, 300 Pasteur Drive Stanford, Stanford, CA 94305 USA; 2 Department of Cardiac Surgery, VA Palo Alto Health Care System, Palo Alto, CA USA; 3 Department of Anesthesiology, VA Palo Alto Health Care System, Palo Alto, CA USA; 4 Department of Anesthesiology, Stanford University Medical Center, Palo Alto, CA USA; 5 Department of Radiology, Stanford University Medical Center, Palo Alto, CA USA

**Keywords:** Quadricuspid aortic valve, Aortic valve replacement, Aortic stenosis, Flail leaflet

## Abstract

**Background:**

Quadricuspid aortic valve (QAV) is a rare congenital anomaly characterized by the presence of four cusps instead of the usual three. It is estimated to occur in less than 0.05% of the population, with Type A (four equal-sized leaflets) accounting for roughly 30% of QAV subtypes. Based on limited clinical series, the usual presentation is progressive aortic valve regurgitation (AR) with symptoms occurring in the fourth to sixth decade of life. Severe aortic valve stenosis (AS) and acute AR are very uncommon.

**Case presentation:**

We describe two cases of Type A QAV in patients who remained asymptomatic until their seventies with very uncommon presentations: one with severe AS and one with acute, severe AR and flail leaflet. In Case A, a 72-year-old patient with history of moderate AS presents to clinic with progressive exertional dyspnea. During work-up for transcatheter vs. surgical replacement pre-operative computed tomography angiogram (CTA) reveals a quadricuspid aortic valve with severe AS, and the patient undergoes surgical aortic valve replacement. Pre-discharge transthoracic echocardiography (TTE) shows good prosthetic valve function with no gradient or regurgitation. In Case B, a 76-year-old patient is intubated upon arrival to the hospital for acute desaturation, found to have wide open AR on catheterization, and transferred for emergent intervention. Intraoperative TEE reveals QAV with flail leaflet and severe AR. Repair is considered but deferred ultimately due to emergent nature. Post-operative TTE demonstrates good prosthetic valve function with no regurgitation and normal biventricular function.

**Conclusions:**

QAV can present as progressive severe AS and acute AR, with symptoms first occurring in the seventh decade of life. The optimal treatment for QAV remains uncertain. Although aortic valve repair or transcatheter option may be feasible in some patients, aortic valve replacement remains a tenable option.

**Supplementary Information:**

The online version contains supplementary material available at 10.1186/s13019-024-02696-w.

## Background

Quadricuspid aortic valve (QAV) is a rare congenital defect characterized by the presence of four cusps, instead of the usual three [1, 2]. It is estimated to occur in < 0.05% of the population and often presents as progressive aortic valve regurgitation (AR) in the fourth to sixth decade [1, 2]. Aortic valve stenosis (AS) is uncommon. We report two patients who remained asymptomatic until their seventies and underwent successful surgical valve replacement—one for severe AS and one for acute, severe AR. Informed consent was obtained from both patients.

## Case Presentation

### Case A

**Fig. 1:**
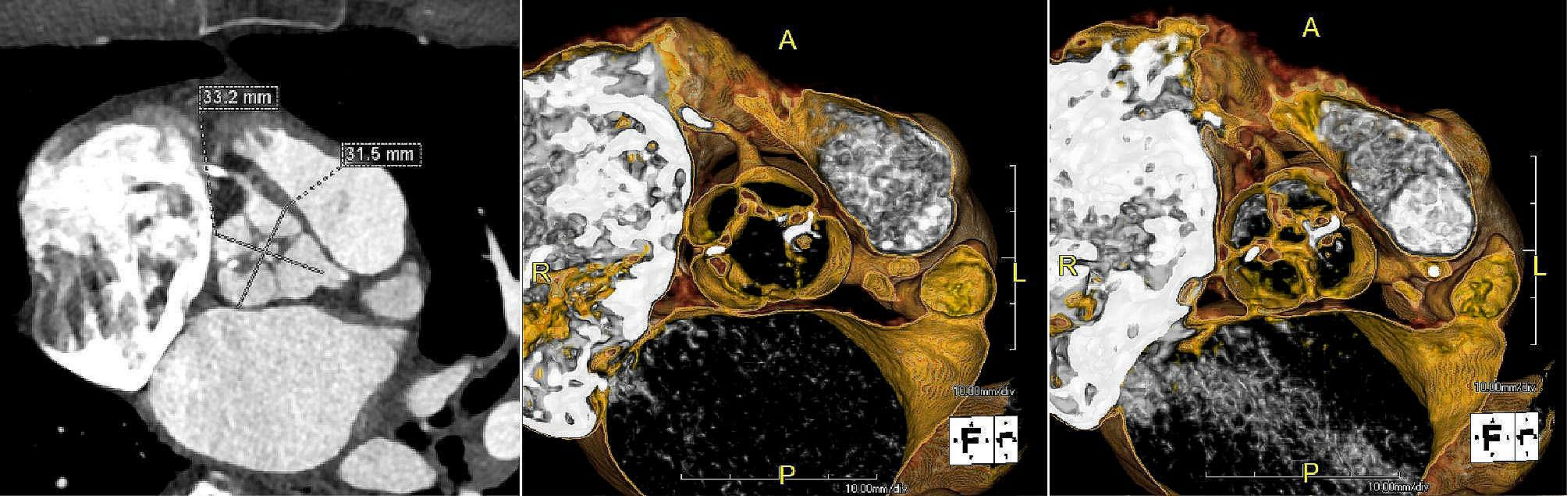
Pre-operative Computed Tomography of Quadricuspid Aortic Valve – Case A. (Left) 2-dimensional slice depicting quadricuspid aortic valve *en face*. The annular diameter was measured at 32 × 33 mm, and the cusps appeared equal in size. The origins of the left and right coronary arteries can be seen in their usual anatomic position. (Middle) 3-dimensional reconstruction of the valve in systole. Asymmetry of leaflet mobility is apparent and is likely due to calcification/fibrosis that is causing physiologic stenosis. (Right) 3-dimensional reconstruction of the valve in diastole. Central area of non-coaptation can be appreciated, as well as the equal size of the leaflets

A 72-year-old man with history of hypertension, left carotid artery stenosis, and stage 1 lung adenocarcinoma status post elective radiation presented to multidisciplinary valve clinic for several months of progressive dyspnea in the setting of severe AS and moderate AR. During his pre-operative lung cancer work-up a year prior, TTE showed mean valve gradient 33 mmHg and ejection fraction 58%. Repeat TTE recently showed mean gradient 46 mmHg with aortic valve area 0.89 cm [2] and moderate AR, and the patient was referred for evaluation for transcatheter vs. surgical replacement. As part of transcatheter work-up, detailed pre-operative computed tomography angiogram (CTA) was obtained and revealed a quadricuspid aortic valve with normal coronary anatomy (Fig. 1). Electrocardiogram at clinic visit showed new diagnosis of atrial fibrillation. Notably, he was still working as a laborer four days a week at this time. Due to poor femoral access, severe carotid artery stenosis, and otherwise good surgical candidacy, the patient was recommended to undergo surgical aortic valve replacement (SAVR) with modified MAZE procedure.

**Fig. 2:**
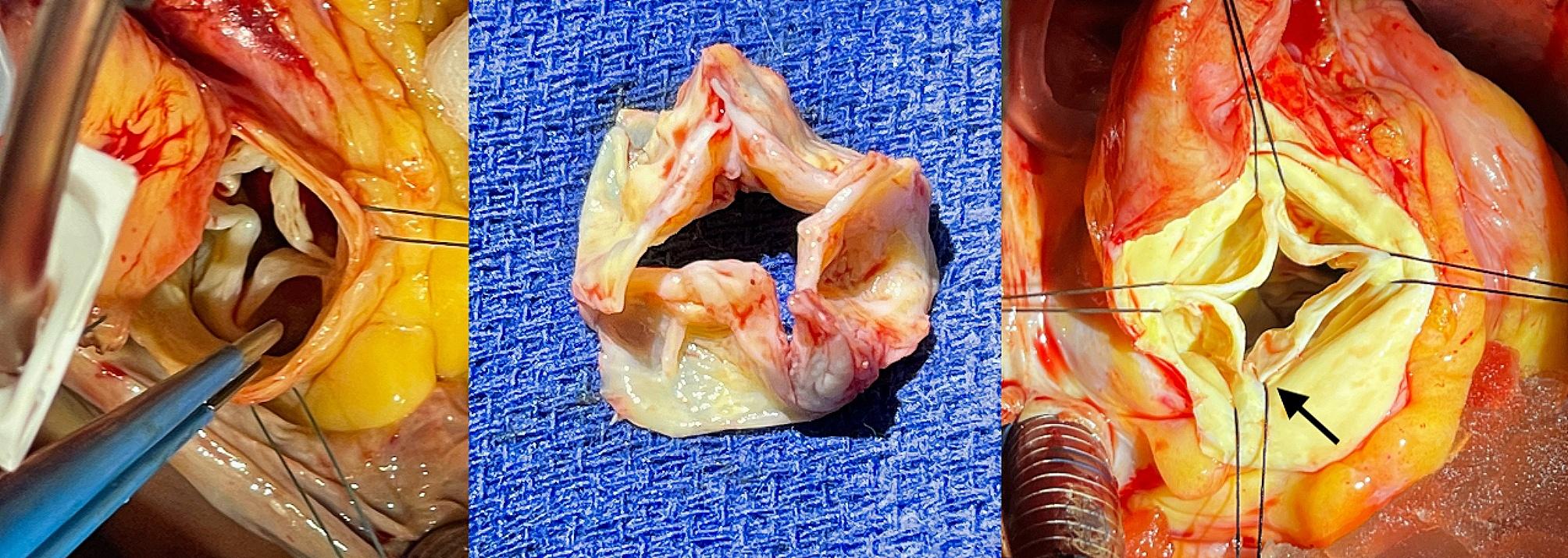
Intra-operative Pictures of Quadricuspid Aortic Valve. (Left) Quadricuspid aortic valve in-situ – Case A. (Middle) Explanted quadricuspid aortic valve – Case A. (Right). Quadricuspid aortic valve in-situ – Case B

He underwent SAVR with a 23 mm bioprosthetic valve, with visual confirmation of four equally-sized leaflets and normal coronary relation (Fig. 2). The patient was extubated on post-operative day (POD) 0 and transferred out of the intensive care unit on POD3. Due to persistent atrial fibrillation he was cardioverted on POD5 and discharged home on POD12 in sinus rhythm. Pre-discharge TTE showed good prosthetic valve function with no residual gradient or regurgitation.

### Case B

A 76-year-old man with history of hypertension, hyperlipidemia, and emphysema was brought in by ambulance in the middle of the night for sudden shortness of breath found to be in acute pulmonary edema with oxygen saturation of 80%. Electrocardiogram showed possible ST changes in leads V1-3. Cardiac catheterization revealed flail aortic valve leaflet with severe AR and preserved systolic function (supplemental video 1). The patient was transferred for emergent valve surgery. Intraoperative TEE was notable for QAV with severe AR and flail leaflet (supplemental video 2). Under direct visualization, there were four equal-sized leaflets, with multiple fenestrations in the valve and prolapse (Fig. 2). The patient underwent SAVR with a 23 mm bioprosthetic valve and had an uneventful post-operative course. He was discharged to skilled nursing facility on POD10. Post-operative TTE showed good prosthetic valve function with no regurgitation.

## Discussion and conclusions

QAV is a rare congenital anomaly often associated with progressive symptoms of AR. Its embryologic etiology is not known, but some have attributed it to aberrant fusion of the aortopulmonary septum or abnormal proliferation in common trunk [3]. According to the Hurwitz and Roberts classification of semilunar valves, there are seven types of QAV based on relative size of the four cusps [3]: type A (four equal cusps), type B (three equal cusps, one smaller cusp), type C (two equal cusps larger than two other equal cusps), type D (one large, two intermediate, one small cusp), type E (three equal cusps, one larger cusp), type F (two equal cusps larger than two unequal cusps), type G (four unequal cusps). Both patients presented had type A QAV (four equal-sized cusps).

QAV can be diagnosed by echocardiography, CTA, or magnetic resonance imaging. The natural history of QAV is poorly defined, as most reports represent small single-center experiences. The largest retrospective study-to-date reported 50 cases over a 39-year period [1]. It found QAV was more common in males (52%) and diagnosed at mean age of 43.5 years. The most common types of QAV were type A (32%) and type B (32%). Moderate or severe AR was diagnosed in 26% of patients, while stenosis affected only 8% of valves and was mild. During mean follow-up of 4.8 years, eight patients underwent valve surgery, with severe AR being the indication in seven patients. Regarding associated cardiac and coronary anomalies, they reported coexisting structural cardiac anomalies in 32% and evidence of aortic dilation in 29% of their patients. There are additional isolated reports of coexistent atrial or ventricular septal defect, patent ductus arteriosus, tetralogy of Fallot, mitral valve prolapse, hypertrophic cardiomyopathy, or anomalous origin or epicardial distribution of coronary arteries, but the majority of QAV cases are isolated [4–9].

Although positional variation of the conduction system has not been identified, several reports suggest that when the supranumerary leaflet is located above the membranous septum there may be closer proximity between the conduction system and the inferiorly displaced leaflet attachment and therefore higher risk of post-operative heart block [10, 11]. These authors go on to propose modified implantation techniques that avoid sutures below the valvar hinge point such as supraannular placement.

The cases presented appear unique in their delayed presentation and the type of valvular pathology—one with severe AS and one with acute, severe AR. Given the rarity of severe stenosis, prior radiation may have contributed in Case A but only in part, as moderate AS was already present. Although there were no signs of endocarditis in Case B, it may have been subclinical given fenestrations noted on the valve. There are no studies showing correlation between QAV type and timing of presentation, but Type A may be predisposed to later presentation given leaflet symmetry.

The optimal treatment for QAV is not well established, but surgical intervention is indicated for symptomatic, severe AS or AR. Liu et al. reported a series of five patients who underwent TAVR, with good short-term outcomes for four patients with severe AR, but the patient with severe AS required permanent pacemaker for new heart block [12]. Aortic valve repair may be feasible for some patients with well-preserved cusps [7]. However, most patients require aortic valve replacement due to the complex anatomy and dysfunction of QAV.

### Electronic supplementary material

Below is the link to the electronic supplementary material.

Supplementary Material 1

Supplementary Material 2

## Data Availability

Data supporting the results reported can be found in the electronic health records from Stanford University Medical Center and VA Palo Alto Healthcare System, and are available from the corresponding author on reasonable request.

## Abbreviations

QAV: Quadricuspid aortic valve
AS: Aortic stenosis
AR: Aortic regurgitation
TEE: Transesophageal echocardiogram
TTE: Transthoracic echocardiogram
CTA: Computed tomography angiogram
SAVR: Surgical aortic valve replacement
TAVR: Transcatheter aortic valve replacement
